# A COMMUNITY-UNIVERSITY PARTNERSHIP TO ASSESS AFRICAN IMMIGRANT FAMILIES' EXPERIENCES WITH DEMENTIA

**DOI:** 10.1093/geroni/igac059.239

**Published:** 2022-12-20

**Authors:** Manka Nkimbeng, Christina Rosebush, Kwame Akosah, Hawking Yam, Wynfred Russel, Elizabeth Albers, Tetyana Shippee, Joseph Gaugler

**Affiliations:** University of Minnesota, Minneapolis, Minnesota, United States; University of Minnesota, Minneapolis, Minnesota, United States; University of Minnesota, Minneapolis, Minnesota, United States; University of Minnesota, Minneapolis, Minnesota, United States; African Career Education And Resources Inc. (ACER), Brooklyn Park, Minnesota, United States; University of Minnesota School of Public Health, Minneapolis, Minnesota, United States; University of Minnesota, Minneapolis, Minnesota, United States; University of Minnesota, Minneapolis, Minnesota, United States

## Abstract

African immigrants are a fast-growing segment of the U.S. Black population, but the dementia care needs and resources of this population are not fully understood. We will describe the process of developing culturally informed instruments to collect data on dementia care needs and resources among African immigrants. Working together with a diverse project advisory board, a guide was developed and used to conduct community conversations about experiences with dementia/memory loss. Qualitative findings from these conversations were used to inform the development of a survey for quantitative data collection. Despite the challenges of conducting research during a global pandemic, having trusting relationships with a partnering community organization and project advisory board facilitated the successful development of instruments to conduct preliminary dementia care research in an underserved population. We anticipate that survey results will inform interventions that increase education, outreach, and access to dementia care and caregiving resources for this population.

